# Nanomaterial Modification of Ultramicroelectrodes Using Design-of-Experiments Principles

**DOI:** 10.1021/acselectrochem.5c00227

**Published:** 2025-11-27

**Authors:** Rachel A. Bocking, Thomas M. Dixon, Brenna Parke, Parastoo Hashemi, Richard A. Bourne, Paolo Actis, Robert Menzel

**Affiliations:** 1 School of Chemistry, 4468 University of Leeds, Leeds LS2 9JT, United Kingdom; 2 Bragg Centre for Materials Research, 4468 University of Leeds, Leeds LS2 9JT, United Kingdom; 3 Institute of Process Research and Development, 4468 University of Leeds, Leeds LS2 9JT, United Kingdom; 4 Department of Bioengineering, 4615 Imperial College London, London SW7 2AZ, United Kingdom; 5 School of Electrical and Electronic Engineering, 4468 University of Leeds, Leeds LS2 9JT, United Kingdom

**Keywords:** ultramicroelectrode, electrophoretic deposition, design of experiments, H_2_O_2_, sensing, nanomaterials

## Abstract

Modification of ultramicroelectrode sensors with electroactive nanomaterials is key to enhancing their microscale sensing performance for advanced applications in cellular biology, disease diagnostics, or scanning electrochemical microscopy (SECM). This work employs a modern design-of-experiment (DoE) approach to develop a systematic, multiple-parameter methodology for the development of robust ultramicroelectrode modification protocols. Specifically, platinum ultramicroelectrode sensors are coated with platinum/nanocarbon nanocomposites through electrophoretic deposition (EPD), using 2^*k*^ factorial screening designs to systematically investigate the ultramicroelectrode modification process. The steady state current is employed as a quantitative DoE target metric, enabling us to map and model optimum ultramicroelectrode modification conditions. DoE-optimized modification conditions are shown to achieve substantial improvements in coating quality and limit of detection in a model H_2_O_2_ sensing study. The DoE-optimized conditions are also successfully translated to the modification of carbon-fiber ultramicroelectrodes (CFM), achieving effective modification in a single experiment. This systematic DoE approach provides a versatile, robust, and highly effective method for developing ultramicroelectrode modification across multiple parameters through a minimal number of experiments. Importantly, the DoE methodology also readily identifies tolerances and limiting conditions for the modification process, vital for broader adoption and future technology translation of functionalized ultramicroelectrodes.

## Graphical abstract

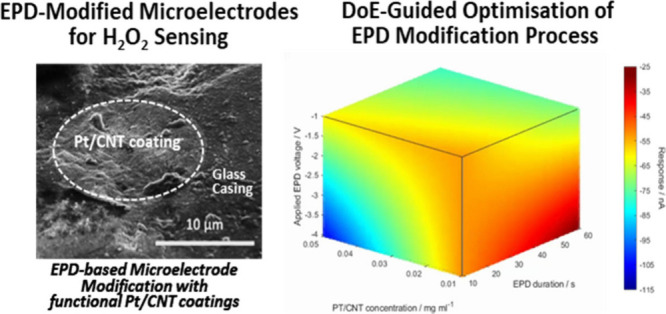

## Introduction

Ultramicroelectrodes, defined as having one or more dimensions less than 25 μm,^1^ have been extensively employed for molecular sensing applications as they pair small electrode sizes with sensitive detection and superior spatial resolution, making them attractive for biomedical applications and for integration into electrochemical scanning probe technologies.^2–6^ Modification of ultramicroelectrode surfaces through coating with functional nanomaterials or molecular sensitizers is an important strategy for the enhancement of sensing sensitivity and limit of detection (LOD). Examples include ultramicroelectrode functionalization with metallic nanoparticles, enzymes, polymers, or dyes to increase selectivity towards a specific analyte.^7–13^ Another example relevant to this work includes the coating of ultramicroelectrode surfaces with carbon nanomaterials, such as carbon nanotubes (CNTs), to increase the available electroactive surface area, improving electron transfer between the analyte and electrocatalyst. Such modified ultramicroelectrodes have been explored in electrochemical sensing systems for a diverse array of applications, from biological sensing to energy storage.^14–18^ For the creation of such functional nanomaterial coatings, electrophoretic deposition (EPD) has been shown to be a particularly useful technique. EPD-based electrode modification is centred on the application of an electrical potential through a colloidal suspension of charged nanoparticles to form a particle coating via electrophoresis and electrodeposition.^19,20^ As such, EPD provides a rapid, cheap, and highly versatile methodology for electrode modification with functional nanomaterials. Consequently, EPD has been used frequently for coating macroscale electrode surfaces and has also considerable potential for use in coating micro and nanoscale electrode tips.^19^

However, robust and reproducible modification of ultramicroelectrode surfaces via EPD can be difficult to achieve, as even minor inhomogeneities in the nanoparticle coating can have a detrimental impact on sensor performance.^21^ As highlighted by Weber et al., a fine balance between electrode modification parameters is required to achieve adequate coating formation.^13^ Optimization of the EPD process is typically needed to improve the ultramicroelectrode coating quality. Such optimization is conventionally carried out via one-variable-at-a-time (OVAT) approaches, which are time-consuming and can overlook important interrelationships between multiple parameters (thereby potentially missing global optima). Additionally, the evaluation of coating homogeneity after each optimization step is challenging for ultramicroelectrodes as the ultrasmall electrode dimensions typically require challenging electron microscopy imaging, which is lengthy, expensive, and can damage the deposited layer. To address these issues, this work explores an alternative approach based upon design of experiments (DoE) principles as a systematic, data-driven method to find optimized ultramicroelectrode modification conditions with a minimal number of experiments.

DoE-based optimization methodologies have proven highly successful in chemical process engineering^22,23^ but have not yet been explored in the context of ultramicroelectrode modification. DoE approaches are based on the systematic exploration of process conditions across a design space, followed by the analysis of the corresponding process response through well-established mathematical models.^24–26^ As such, DoE allows for the interactions between parameters to be investigated and identifies optimal process conditions, promoting process efficiency and repeatability. DoE approaches, therefore, have great potential for the development of robust, repeatable ultramicroelectrode modification methods. Specifically, DoE enables simultaneous exploration of multiple modification parameters in a highly systematic fashion to determine the best ultramicroelectrode modification conditions as well as process tolerances in a minimal number of experiments.

To demonstrate these benefits, this work applies DoE principles to efficiently optimize ultramicroelectrode modification with functional nanoparticle coatings. Specifically, this work studies the modification of commercial platinum ultramicroelectrodes (Pt-ME) with a platinum-nanoparticle/carbon-nanotube (Pt/CNT) composite (Figure 1a), with the aim to improve the electrochemical sensing of H_2_O_2_, an important analyte in the study of cellular metabolism^27,28^ and disease diagnostics.^29–32^ Modification of the ultramicroelectrode surface with the Pt/CNT composites is carried out via EPD (Figure 1b) and optimized via variation of three key EPD parameters (EPD voltage, EPD duration, and Pt/CNT concentration in the EPD bath). A key objective is to establish a systematic and resource-conscious DoE optimization methodology through an experimental DoE framework that allows to minimize the number of required modification and characterization experiments, thereby reducing the consumption of ultramicroelectrodes. To this end, a 2^3^ design space is explored (Figure 1c), using an electrochemical current as a quantitative response metric for coating quality. The resulting output data are fitted to a simple mathematical interaction model. Further refinement of the model is achieved through a simplified two-factor DoE study. To demonstrate the success of this DoE strategy, the Pt/CNT-modified Pt-MEs are assessed in terms of electrochemical H_2_O_2_ sensing (LOD and sensitivity). The optimized modification parameters are then shown to be readily translatable to different ultramicroelectrode systems, such as carbon fiber ultramicroelectrodes (CFMs), highlighting the versatility of DoE-based optimization.

**Figure 1:**
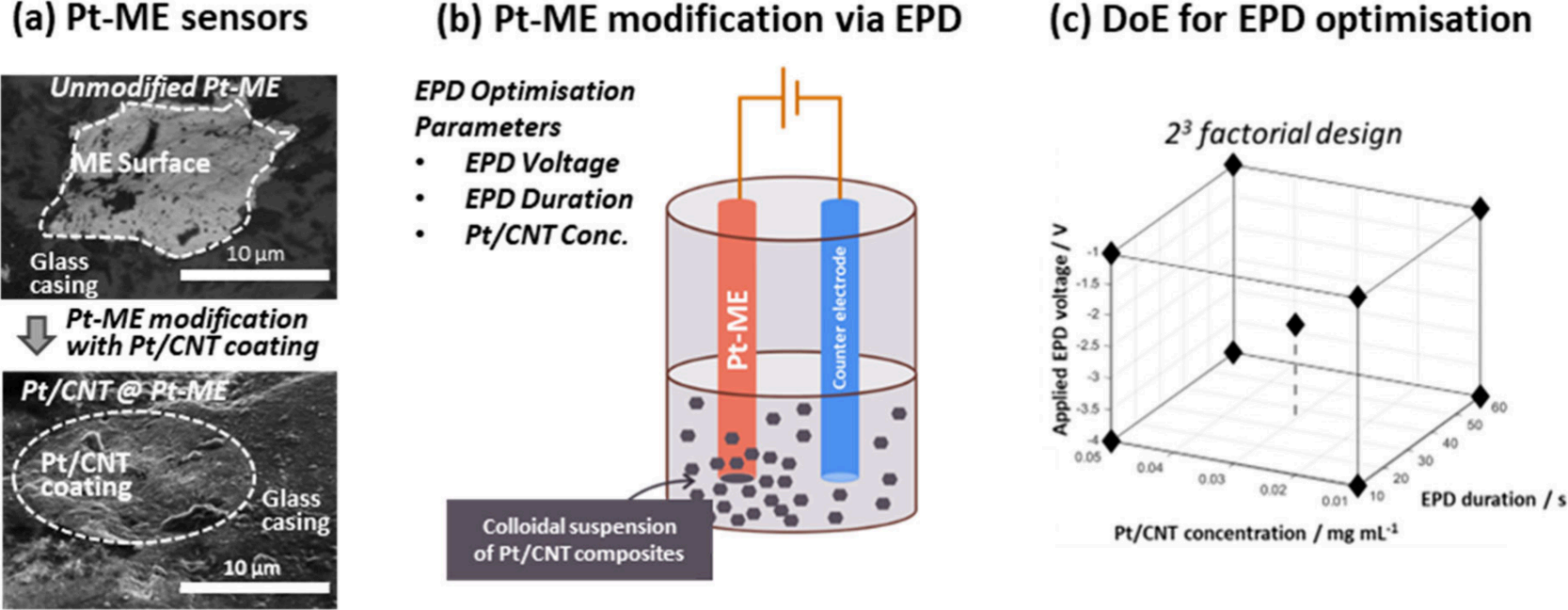
Ultramicroelectrode modification optimization via a design of experiments (DoE) approach: (a) SEM images of the microscale electrode surface of an unmodified Pt-ME surface (top) and a Pt-ME surface coated with Pt/CNT electrocatalysts; (b) working principle of electrophoretic deposition (EPD), used to carry out ultramicroelectrode modification with Pt/CNT coatings (N.B. in practice a three electrode configuration is used; the reference electrode is omitted in the figure for clarity); (c) DoE parameter space, used to optimize Pt-ME modification via EPD.

## Experimental Section

### Materials and Equipment

Acid-oxidized multiwalled CNT, H_2_PtCl_6_ (8 wt % in solution), NaOH pellets, HPLC grade water, dimethylformamide (DMF), and ethanol (absolute) were purchased from Sigma-Aldrich. NaBH_4_ was purchased from Fisher Scientific. For electrochemical measurements, a VSP potentiostat (Biologic) was used with a Faraday cage to shield electromagnetic noise. Data acquisition and analysis were performed by using EC Lab software. Pt-MEs were purchased from Biologic (model U-23/15, 15 μm diameter). The Ag/AgCl reference electrode was purchased from VWR. A Pt wire was used as the counter electrode. CFMs were provided by the Hashemi group (Imperial College London). pH 7.4 10× phosphate buffer solution (PBS) was purchased from Gibco and diluted using HPLC-grade water. Ru­(NH_3_)_6_Cl_3_ was purchased from Sigma-Aldrich and diluted in KCl. H_2_O_2_ (30 w/w%), purchased from Merck.

### Pt/CNT Synthesis

Pt/CNT was synthesized using a chemical reduction method adapted from work by Li et al.^33^ Acid-oxidized multiwalled CNTs (20 mg) were first dispersed in HPLC-grade water (10 mL) using probe tip sonication (GEX130 ultrasonic processor, 130 W, 20 kHz) for 20 min at 30 % amplitude. A further 90 mL of HPLC-grade water was then added. Under stirring, H_2_PtCl_6_ (100 μL) was added to form a suspension at pH 5.3. The suspension was adjusted to pH 10 using NaOH. Following this, NaBH_4_ (800 mg) was slowly added as a reductant. The stirring mixture was left at room temperature for 24 h. The solid product was then removed by vacuum filtration and washed with excess ethanol and HPLC-grade water. The resulting composite was then freeze-dried for 48 h (Labconco Freezone −50 °C freeze dryer).

Electrodeposition of Pt nanoparticles (used to produce the ultramicroelectrodes labeled as Pt_(ED)_/CNT@Pt-ME; Pt_(ED)_/CNT@CFM; and Pt@CFM in the main text) was carried out, using a method reported by Actis et al.^34^ Specifically, ultramicroelectrodes were mounted within the electrochemical setup described below, and Pt nanoparticle electrodeposition was carried out by sweeping a potential from 0 to −800 mV at 200 mV/s vs Ag/AgCl in 2 mM H_2_PtCl_6_ in 0.1 M HCl.

### Modification of Ultramicroelectrodes via EPD

EPD coating of Pt/CNT composites and the multiwalled CNT only onto the ultramicroelectrodes was carried out in a glass cell, using a 3-electrode setup (BioLogic VSP potentiostat with EC Lab software, using an Ag/AgCl reference electrode and Pt wire counter electrode), using a constant voltage method for the corresponding applied voltage and EPD duration in the DoE design space. Prior to EPD experiments, all Pt-MEs were cleaned by rinsing in deionized water, polishing with 0.3 μm alumina slurry (Metrohm polishing kit), followed by rinsing in deionized water and then bath sonication for 10 min in a 1:1 ethanol and water mixture. To prepare the specified concentrations of suspended Pt/CNT and CNT in the EPD bath, to the corresponding mass of solid was added DMF (5 mL). This was then dispersed using probe tip sonication at 30 % amplitude for 10 min.

DMF was selected as the solvent for the EPD experiments due to the least aggregation and flocculation of both Pt/CNT and unmodified CNT. DMF has also been used as a solvent for CNTs in other studies.^21^ Previous work has highlighted EPD condition ranges of 5–50 V and 0.5–10 min for the deposition of CNTs onto stainless steel macroelectrodes.^35^ However, for microscale electrodes, the tips are delicate and fragile, meaning they cannot tolerate high voltages, and thick, dense coatings are not required. Therefore, milder conditions were chosen for the EPD of Pt/CNT composites onto the Pt-MEs.

To produce the prototypical coatings shown in Figure 2, Pt-MEs were modified via EPD from dispersions of presynthesized Pt/CNT in DMF at −4 V, 0.1 mg/mL Pt/CNT, 300 s (Figure 2(a-i) and at −4 V, 0.05 mg/mL Pt/CNT, 10 s (Figure 2(b-i), respectively. The coating in Figure 2(c) was produced through drop casting of a 0.01 mg/mL CNT/Pt dispersion in DMF onto the Pt-ME, followed by vacuum oven drying (because it was difficult to achieve monolayer Pt/CNT coating via EPD prior to the optimization study). Drop casting was attempted several times and gave very variable coatings; from SEM imaging, the coating most closely resembling thin, monolayer formation was selected for electrochemical characterisation.

**Figure 2:**
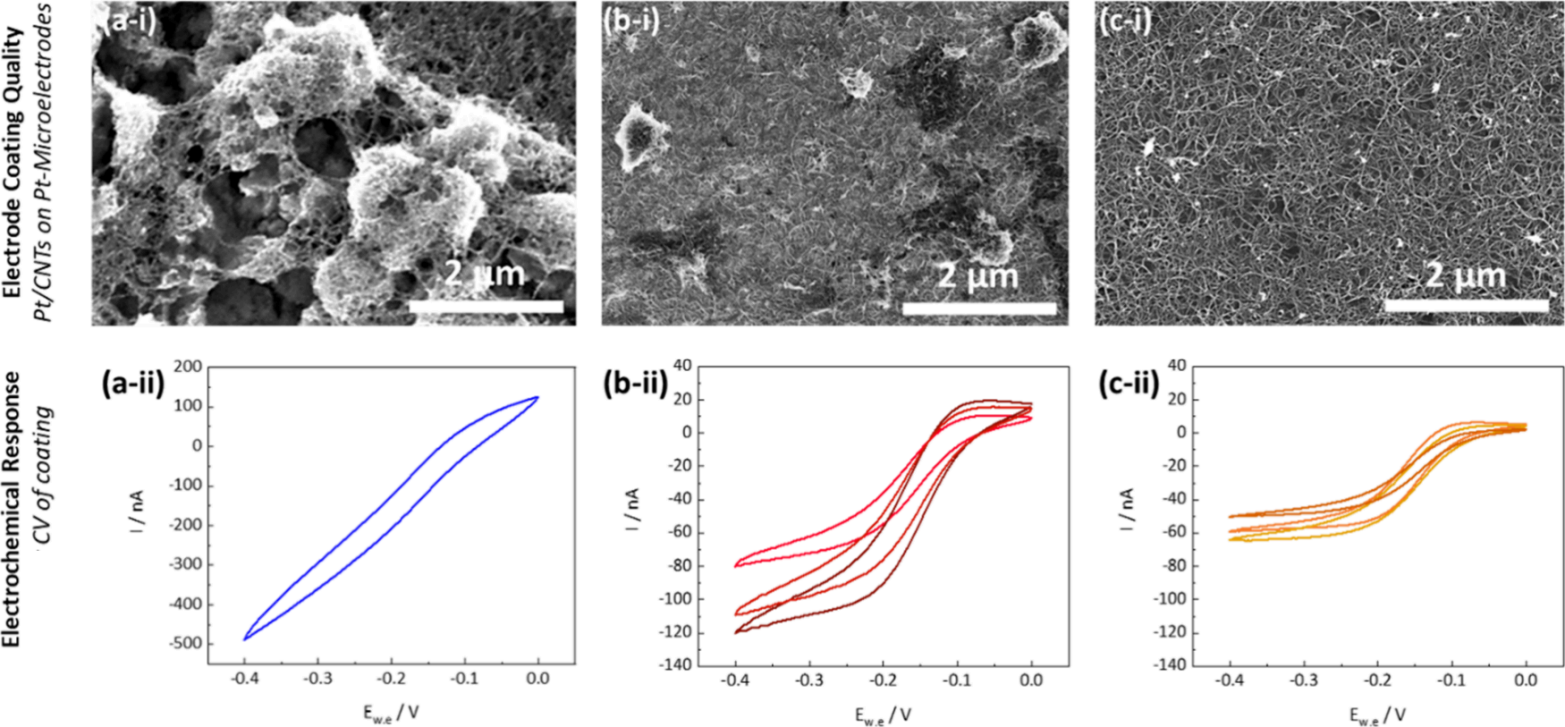
SEM images (indicating ultramicroelectrode coating quality) and their respective Ru­(NH_3_)_6_Cl_3_ CV curves at 100 mV/s (indicating deviation from ideal ultramicroelectrode electrochemical response): (a-i, a-ii) thick, multilayer coating of Pt/CNT associated with pronounced capacitive charging currents and loss of typical sigmoidal ultramicroelectrode response shape (coating produced via EPD at *V* = −4 V, *C* = 0.1 mg mL^–1^; *D*_t_ = 300s) ; (b-i, b-ii) medium thickness Pt/CNT coating with some agglomerations of CNTs which lead to inhomogeneities and sigmoidal but less repeatable *I* measurements (coating produced via EPD at *V* = −4 V, *C* = 0.1 mg mL^–1^; *D*_t_ =10 s); (c-i, c-ii) thin, homogeneous Pt/CNT coating on Pt-ME, leading to an optimal *I* of −55 ± 5 nA (coating produced via drop casting from dispersion, *C* = 0.01 mg mL^–1^).

### DoE Methodology and Model Fitting

An interaction model was applied to the data set to assess the correlation between EPD factors. This model is represented by eq 1 for the 2^3^ design space and eq 2 for the 2^2^ design space. The *X_n_* terms represent the constants (where *n* is the labeled constant number) which dictate the value of *I* based on the three factors, *C*, *V*, and *D*_*t*_ (as defined in the main text as concentration of Pt/CNT in the EPD bath, EPD voltage, and EPD duration, respectively). Least squares regression was used to fit the data to the interaction model and determine the value of the constants. The quality of the fit was assessed using statistical parameters, such as the coefficient of determination (*R* ^2^) and the RSME.

1 $$I=X_{0}+X_{1}V+X_{2}D_{t}+X_{3}C+X_{4}VD_{t}+X_{5}VC+X_{6}D_{t}C$$

2 $$I=X_{0}+X_{1}D_{t}+X_{2}C+X_{4}D_{t}C$$

The code for the DoE and model fitting was written in MATLAB and is available via GitHub.^36^

### Electrochemical Characterization of Ultramicroelectrodes

To determine the steady current value (*I*), CV was carried out in 10 mM Ru­(NH_3_)_6_Cl_3_ in 0.1 M KCl and the current at −400 mV was taken as *I*.^37,38^ The CV method parameters consisted of *E_i_* = 0 V vs Ag/AgCl Ref., scan rate = 100 mV/s (variable), *E*_1_ = −0.4 V vs Ref., *E*_2_ = 0 V vs Ref., nc = 4, 50 % step duration, *N* = 10 voltage steps.

For the detection of H_2_O_2_ in 1× PBS for both Pt-ME and CFM systems, the CV parameters were *E_i_* = 0 V vs Ag/AgCl Ref., scan rate = 100 mV/s (variable), *E*_1_ = 1 V vs Ref., *E*_2_ = −0.5 V vs Ref., nc = 3, 50 % step duration, *N* = 10 voltage steps. Results were collected from the experiments at 100 mV/s, with calibration plots based on the electrochemical current response of the ultramicroelectrodes at the specified H_2_O_2_ concentrations at +600 mV. The electrochemical sensing performance of a modified electrode is determined by its sensitivity to a particular species of interest (nA mM^–1^) and its LOD (μM), the lowest concentration of a measured species that can be reproducibly determined:

3 $$\mathrm{LOD}=\frac{3\sigma I_{\mathrm{blank}}}{\mathrm{sensitivity}}$$

### Carbon Fiber Ultramicroelectrode Fabrication

CFMs were fabricated by the Hashemi group at Imperial College London, using a protocol described by Kawagoe et al.^39^ First, a single carbon fiber (10 μm in diameter) was aspirated into a borosilicate glass capillary (1.0 mm outer diameter, 0.5 mm inner diameter, 10 cm long; A-M Systems, Sequim, WA,USA). A carbon-glass seal was then forged under heat and gravity using a vertical pipette puller (Narishige, Tokyo, Japan). The exposed carbon was cut to 10–20 μm from the carbon-glass seal by using an optical microscope and scalpel. The carbon-glass end of the electrode was dipped in epoxy for 30 s, left to dry overnight, and then cured at 150 and 100 °C for 2 h each. The epoxied electrodes were beveled at 45° using a micropipette beveler (BV-10 beveler, Sutter Instruments, Novato, CA, USA) for ∼1–2 min to expose a smooth carbon-glass surface. Finally, an electrical connection was forged by inserting a pinned stainless-steel wire coated with silver paint into the open end of the capillary.

## Results and Discussion

### EPD-Modified Ultramicroelectrodes for Electrochemical H_2_O_2_ Sensing

Commercially available Pt-MEs were initially selected for modification due to their wide prior applications in electrochemical research.^27,40,41^ Due to their small surface area (approximately 180 μm^2^ for a 15 μm diameter ultramicroelectrode), they show moderate sensitivity towards H_2_O_2_. Ultramicroelectrode performance can be significantly increased through the deposition of nanometer-sized, high-surface-area platinum nanoparticles. Alone, platinum nanoparticles tend to sinter into undesirable, large, low-surface area aggregates; however, this is often mitigated by supporting them on conducting high-surface-area carbon nanostructures such as CNTs.^42,43^ Here, Pt/CNT composites were formed between Pt nanoparticles and multiwalled CNTs to prevent low-surface area platinum aggregates. The Pt/CNT composites were produced in-house and consisted of Pt nanoparticles (∼4.8 nm in diameter) loaded at ∼10 wt % onto CNTs (Figures S1 and S2). The preformed Pt/CNTs were readily dispersible in DMF to form time-stable Pt/CNT dispersions. Pt-MEs were then coated with the pre-formed Pt/CNT composites via EPD from the Pt/CNT dispersions, with the aim to improve H_2_O_2_ sensing LOD.^44^

An important reason for selecting Pt-MEs as the initial model system is their commercial availability and superior mechanical durability compared to those of many other ultramicroelectrode types. This robustness facilitates the development of DoE-based modification strategies that require multiple electrodeposition experiments under varying conditions. Pt-MEs can be cleaned after each experiment via polishing, enabling their reuse in subsequent modification trials.^1^ However, the inherent variability of commercial ultramicroelectrodes, combined with repeated use across experiments, can result in surface irregularities (Figure 1a). These irregularities may influence electrodeposition outcomes and introduce experimental variability. Importantly, the primary aim of this study was to develop and validate a deposition optimization strategy capable of predicting broad trends, even when applied to ultramicroelectrodes with non-ideal surface morphologies. While surface defects and geometric imperfections are expected to contribute to greater variability, the robustness of the DoE-based protocol explored in this work is demonstrated by its success in optimising sensor performance, as detailed in later sections. The versatility and reliability of the DoE strategy are further underscored by its successful application to more fragile, glass-encased disk CFMs, as presented in the final section.

### DoE Design Space for EPD-based Ultramicroelectrode Modification

To define the initial design space for the EPD-based modification of Pt-MEs with Pt/CNT, the EPD deposition time (*D*_*t*_), the applied EPD voltage (*V*), and the concentration of suspended Pt/CNT modification agent (*C*) were selected as key parameters, based on prior literature.^13,45,46^ To form the design space, specific upper and lower bounds for the three DoE factors needed to be selected. The boundaries for *V* were selected to be large enough to overcome repulsive interactions that prevent EPD (lower bound −1 V) but not too large to damage the Pt-ME (upper bound −4 V).^47^ The concentration of the Pt/CNT composites was kept below 0.1 mg/mL to maximize suspension stability and minimize agglomeration (upper bound 0.05 mg mL^–1^)^21^ but was kept high enough to ensure reasonable deposition rates (lower bound 0.01 mg mL^–1^). Deposition time boundaries were chosen based on initial experiments. *D*_*t*_ values less than 10 s were eliminated as this was insufficient to produce any coating (as evidenced by scanning electron microscopy (SEM), Figure S3), while values larger than 60 s were excluded as this led to relatively thick deposits with poor adhesion to the ultramicroelectrode surface.

To systematically explore ultramicroelectrode modification within this parameter space, a simple 2^*k*^ factorial design was employed, where *k* is the number of experimental parameters explored (*V*, *D*_*t*_, and *C*). Specifically, the selected 2^3^ DoE design explored the extremities of the EPD parameter space, with an additional experiment at the center of the design space (Figure 1c). This design was selected as it is a commonly used screening design and requires a relatively small number of experiments.^48^ A simple screening DoE was selected over the alternative DoE designs (such as more complex response surface methodology designs or Taguchi arrays)^24,49^ to minimize the number of experiments (with each experiment requiring a new Pt ultramicroelectrode). Specific data points were repeated to enable measurement of the variance within the design space. Two repeat experiments were carried for a corner point (rather than at the center of the design space), specifically at high concentration conditions where prior experimental experience had shown that Pt-ME modification varied particularly strongly. This led to a total of 11 experiments across the EPD design space (see Table 1 for detailed conditions).

**Table 1:** Nine Combinations of Experimental Conditions Make Up the Design Space for the Three-Factor DoE Design, with Two Repeats at One Set of Conditions, Resulting in 11 Experiments

experiment number	applied EPD voltage, V	EPD duration, s	Pt/CNT concentration, mg mL^–1^
1	–4	10	0.01
2	–4	10	0.05
3	–4	60	0.01
4-6	–4	60	0.05
7	–1	10	0.01
8	–1	10	0.05
9	–1	60	0.01
10	–1	60	0.05
11	–2.5	35	0.03

### DoE Response Metric for EPD-Based Ultramicroelectrode Modification

To conduct DoE studies, a robust and quantitative response metric is essential for evaluating the effectiveness of selected EPD parameter combinations in producing high-quality coatings. However, quantifying coating quality on ultramicroelectrodes presents a challenge, as conventional techniques, such as electron microscopy, are time-consuming, costly, and inherently qualitative, making them unsuitable for iterative characterization and mathematical model fitting within the DoE framework. In this work, the electrochemical response of coated ultramicroelectrodes was assessed using the well-established redox mediator hexaammine­ruthenium­(III) chloride (Ru­(NH_3_)_6_Cl_3_). The resulting steady-state current, denoted as *I*, was employed as a quantitative DoE response metric. This current is readily measurable and serves as a proxy for the ultramicroelectrode’s active surface area and coating quality, which is hypothesized to influence its sensing performance. Furthermore, hexaammine­ruthenium­(III) was chosen because of its consistent performance across most electrochemical surface materials and its minimal impact on ultramicroelectrode surface quality (in contrast to alternative redox couples, such as ferrocene methanol, which led to contamination of the ultramicroelectrode surfaces).

To determine *I*, ultramicroelectrodes were modified with Pt/CNT coatings via EPD, followed by measuring their current output at −400 mV by cyclic voltammetry (CV). To gain insight into the correlation between the *I* output metric and the ultramicroelectrode coating quality, CVs were measured for Pt/CNT-modified Pt-MEs with different coating thicknesses (Figure 2). Three distinct coatings were fabricated to exhibit substantial differences in Pt/CNT deposition and thickness, as qualitatively confirmed by scanning electron microscopy (SEM) images (Figure 2(a-i) to Figure 2(c-i)). Complementary quantitative image analysis further revealed marked variations in coating homogeneity, with the thinnest coatings (Figure 2(c-i)) displaying the most uniform and regular porosity (see Supporting Information Figure S4 and Table S1).

For thick multilayer deposit coatings (Figure 2(a-i)), the CV shows relatively large currents (>400 nA) and a clear deviation from the characteristic sigmoidal CV response expected from ultramicroelectrodes (Figure 2(a-ii)). While the larger magnitude of the current indicates a substantially increased electrochemical surface area, the non-sigmoidal shape suggests large capacitive background currents (∼250 nA, see also Supporting Information, Table S1) which likely mask the characteristic hexaammine­ruthenium­(III) redox signal. As such, these multi-layered deposit coatings are unsuitable for sensing applications as the high capacitive charging masks the faradaic currents relevant for sensing.^7,21,50^ For ultramicroelectrode coatings with medium thickness and high density (Figure 2(b-i)), the CVs show a desirable sigmoidal shape (Figure 2(b-ii)) with considerably reduced capacitive current (∼60 nA) that is much more suitable for sensing applications. However, repeated experiments across multiple Pt-MEs indicate a relatively large variability of the steady state current output (*I* = −100 ± 20 nA, *n* = 3, Figure 2(b-ii), potentially due to the presence of a relatively dense Pt/CNT coating with relatively poor porosity (see also Supporting Information, Figure S4, Table S1). For thin, low-density coatings (Figure 2(c-i)), CVs indicate, again, a desirable sigmoidal response (Figure 2(c-ii)), suitable for sensing applications. While the current output is slightly lower, the variability of the steady-state currents is also significantly reduced (*I* = −55 ± 5 nA, *n* = 3) (Figure 2(c-ii)). As shown in the SEM image, a uniform, low-density, highly porous layer of CNTs was observed with an *I* in this range. Statistical SEM image analysis further confirms that this low-density layer represents a coating with the most pronounced and most uniform porosity among the three coatings (Figure S4), likely beneficial for repeatable sensing applications. These findings are in line with literature findings that report excellent sensing performance of CNT mono-layers due to minimal charging currents and significantly improved signal-to-noise ratios.^21^ Based on these observations, *I* = −55 ± 5 nA was selected as the optimum target response for our DoE study. Using Ru­(NH_3_)_6_Cl_3_ current as coating quality proxy and DoE target response metric allows for an overall faster DoE optimization process by minimising reliance on lengthy electron microscopy coating characterisation or determination of full H_2_O_2_ calibration curves for each single modification parameter combination.

### Three-Factor DoE Study

To implement the DoE approach, 11 modification experiments were performed (Table 1, Figure 1) for an initial screening of the EPD parameters. The current of the 11 modified ultramicroelectrodes was then determined, and the responses were plotted with respect to the parameter combinations used, where the color of the points represents the measured value of *I* (Figure 3a, for numerical values, Table S2). Then, to allow predictions of the current response, a mathematical model was fitted to the data (Figure 3b). The response surface can be modeled in different ways including the use of simple linear or quadratic models or more complex nonlinear or Gaussian models.^51,52^ In this instance, a simple interaction model^24^ was applied to the data set to assess the interdependency between the EPD modification parameters and predict the steady-state current at untested areas of the design space. Other models (outlined in the Supporting Information in Figures S5–S7) were considered; however, a trade-off between simplicity and model fit arrived at the model shown here. Least squares regression and root mean squared error (RMSE) were used to quantify the fit of the data, giving the following model for the DoE data set:

4 $$I=-46.1-1.4V+25.5D_{t}-58.3C-46.5VD_{t}+50.2VC+1.7D_{t}C$$

The interaction model (Figure 3b) gave rise to an *R* ^2^ of 0.782 and an RMSE of 18.4. The coefficients in eq 4 reflect the relative contributions of each EPD parameter, *V*, *D*_*t*_, and *C*, on the overall value of the current *I*.

**Figure 3:**
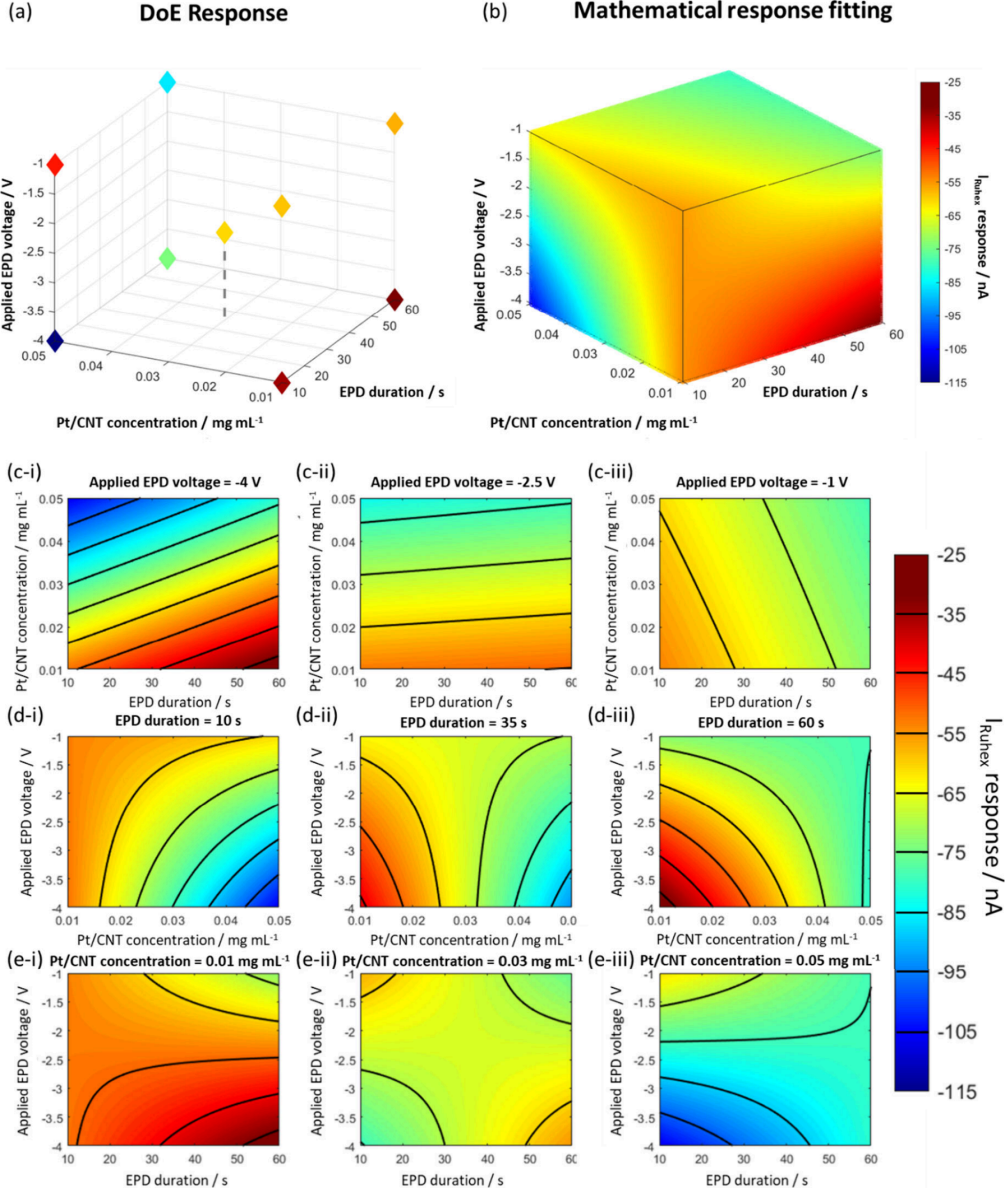
(a) Parameter space of the three-factor DoE design as shown in Figure 1c, here with the points colored to represent their experimental *I* response at each parameter combination; (b) the modeled response surface based on the interaction model (eq 4); (c–e) contour plots, extracted from the modeled 3D *I* response in (b), with the color scale showing predicted variation of *I* (indicative of Pt-ME coating quality) as function of different EPD parameter combinations at constant *V* (c-i, c-ii, and c-iii), constant *D*_*t*_ (d-i, d-ii, and d-iii), and constant *C* (e-i, e-ii, and e-iii), respectively.

The 3D response in Figure 3b can also be represented using 2D contour plots (Figure 3c–e), depicting the (modeled) change of *I* as a function of only two EPD parameters, while at a fixed value of the third EPD parameter (see also Supporting Information Figure S8). The color scale again represents the value of the current *I*. In both the 3D response model and 2D contour plots, orange regions indicate EPD parameter combinations within the target response range of *I* = −55 ± 5 nA, as defined previously, suggesting the formation of low-density ultramicroelectrode coatings, likely associated with good sensing performance. In contrast, red bands indicate responses of I ≈ −30 nA, i.e., in the range of the unmodified Pt-ME response (see Supporting Information Figure S9), indicating that ultramicroelectrode modification at those corresponding EPD parameters is unsuccessful (no interconnected CNT network or no coating at all). Blue bands relate to current responses exceeding −100 nA, suggesting the formation of increasingly thick and less homogeneous ultramicroelectrode coatings. As such, the contour plots aid in the selection of EPD conditions for optimal ultramicroelectrode modification (orange bands). The broadness of the bands reflects the extent to which the current value varies in each plot. Additionally, unfavorable parameter combinations (no coating, or too thick coating) are easily identified (red and blue regions, respectively). For example, in terms of Pt/CNT concentrations, the data indicate that at lower concentrations of around 0.01 mg/mL (Figure 3(e-i)), the region is mostly dominated by optimal coating *I* responses, but there is the possibility of forming minimal coating (red regions). The data also suggest that higher Pt/CNT particle concentrations of 0.05 mg/mL (Figure 3(e-iii)) are increasingly undesirable as they tend to form thicker deposits that can cause larger capacitive charging. These findings are in line with literature findings for the electrodeposition of Ag nanoparticles that demonstrated desirable coatings at short deposition times and lower concentrations.^13^

It should be noted that the relatively small number of repeat experiments and high experimental variability (especially at high concentrations and low voltage) limit the predictive accuracy of the 3D model (eq 4) in certain regions of the design space, e.g., in terms of predicting trends with deposition time. These limitations led us to refine the initial 3D model via a two-dimensional design, described in the next section. However, the simple 3D model provides a useful initial screening framework that allows to deduce broader trends. For example, the coefficients relating to the interactions of *VC* (50.2) and *VD*_*t*_ (46.5) are large, suggesting that their constituent terms are interdependent. This results in curved contour lines in the plots when *V* is varied, representing the interdependence of these variables. In contrast, interdependence between *D*_*t*_ *C* (1.7) is minimal, highlighted by the straighter contour lines in the fixed *V* plots (Figure 3c). Therefore, selecting *V* to be constant would minimize the interactions between factors to produce ultramicroelectrode coatings.

The contour plot where *V* = −4 V (Figure 3(c-i)) shows a wide range of response values with varying *D*_*t*_ and *C*, indicated by narrow contour bands. However, at *V* = −1 V (Figure 3(c-iii)) there is little variation in the *I* response, indicating less scope to optimize *I* at this voltage. From a robustness testing standpoint, choosing regions of the design space that are mostly dominated by optimal *I* values would consequently ensure that any small changes to parameters would result in negligible effects on *I*, leading to greater consistency in subsequent modified ultramicroelectrodes. However, for the scope of this work, it was decided to fix *V* at −4 V to demonstrate current tuneability in a refined two-factor DoE approach (discussed in the next section) and to aid correlation testing between the steady state current and H_2_O_2_ sensing performance (discussed in the final results section) across a broader range of current values.

### Simplified Two-Factor DoE Optimization

Following the three-factor optimization, a simplified two-factor design was explored to further refine the optimal current region across a smaller number of EPD parameters. To demonstrate this, a 2^2^ design was considered, where only *C* and *D*_*t*_ were varied while *V* remained at a fixed value of −4 V (the voltage where the contour plots in Figure 3 showed a wide range of current responses). The 2^2^ design space contained four corner point experiments plus a centre point (Figure 4a). For this work, only one extra experiment was required at the center of the design space, as the corner data points were already collected in the 2^3^ study. Extra repeats were carried out to improve model validity and robustness, resulting in a total of 11 experiments in the new 2^2^ design space (Table S3). The corresponding experimentally measured responses are shown in Figure 4a, while Figure 4b shows the same 2D design space but with the current responses plotted on the *z*-axis for better visualization of current variance for repeat experiments. The two-factor DoE data set was fitted with an interaction model (eq 5, Figure 4c) to give an *R* ^2^ of 0.72 and RMSE of 17.1. The *R* ^2^ for this model decreased slightly compared to that of the 2^3^ design; this is due to the poor repeatability of the data points at high *C* (0.05 mg/mL), especially at low *D*_*t*_. However, the RMSE slightly improved, indicating a more robust model.

5 $$I=-31.1+1.6D_{t}-55.4C+12.2D_{t}C$$

**Figure 4:**
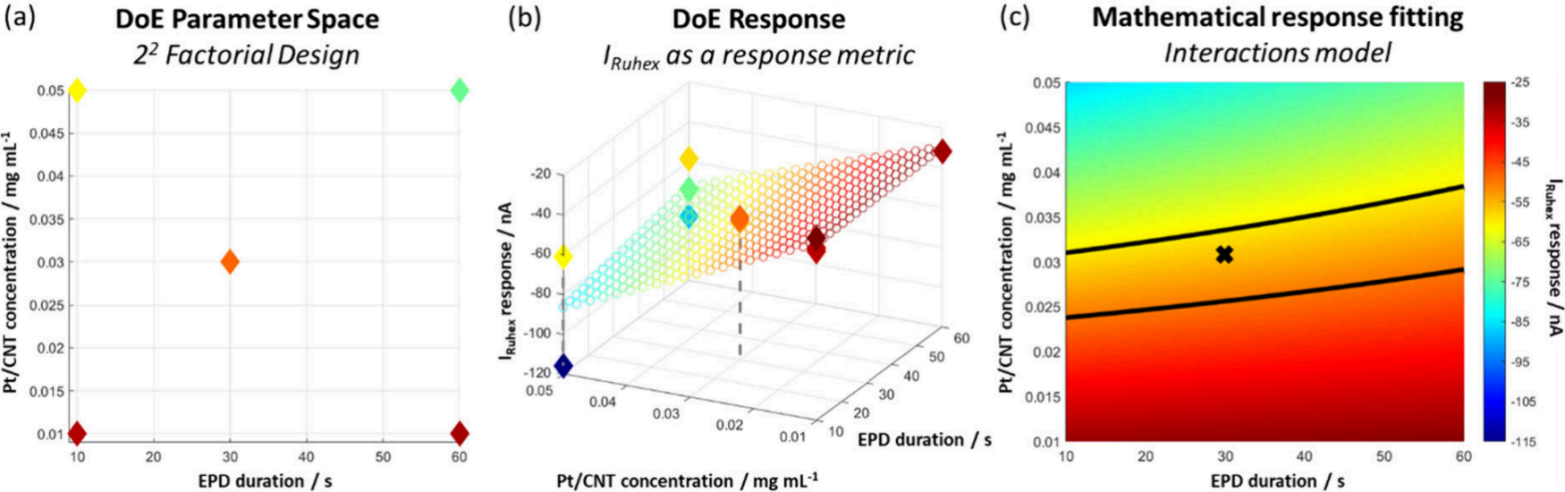
(a) The two-factor design space used for the DoE study with color representing the value of *I*; (b) the same two-factor design space shown in (a) with the current *I* additionally plotted as the *z*-coordinate to show repeatability of data, along with the modeled response surface defined in eq 5; (c) the modeled response surface defined in eq 5 where the region within the black boundary lines highlights suitable parameter combinations that would yield a current response in the region −55 ± 5 nA. The black cross indicates the optimal conditions chosen in this study.

To validate this model, two additional experiments were conducted in unexplored areas of the parameter space (Figure S10). These additional experiments gave a very similar current response to those predicted by the model and an improved *R* ^2^ (0.74) and RMSE (15.1) (Supporting Information Figure S11), highlighting the robustness of the 2D interaction model.

The corresponding modeled 2D response surface (Figure 4c) provides similar insights to the 2^3^ DoE design study where again the data suggest that at very low concentrations EPD modification is unsuccessful (red region), while high Pt/CNT concentrations result in less desirable, thicker deposits (blue region). In contrast, deposition time has only a very minor influence on modification within the 60 s time scale investigated in this work. This highlights the effectiveness of DoE with a limited number of experiments for a multivariable optimization with complex interactions. Overlaying the modeled response surface with the actual measured response data (Figure 4b) indicates that the applied interaction model provides a better fit at lower-to-medium Pt/CNT concentrations. The overlay also illustrates the large spread of data points for repeat experiments at higher concentrations (0.05 mg/mL), further highlighting that low-to-medium Pt/CNT concentrations should be employed to achieve more repeatable ultramicroelectrode modification.

On the fitted 2D response surface in Figure 4c, the optimum EPD parameter space is again indicated by orange color, corresponding to all EPD parameter combinations that are predicted to result in the target current response of −55 ± 5 nA. Ultramicroelectrodes modified using these conditions would likely have enhanced performance in sensing applications due to low-density, highly porous monolayer coatings. Based on these findings, for subsequent testing in H_2_O_2_ sensing, ultramicroelectrodes were modified at an optimized EPD parameter combination of *V* = −4 V, *C* = 0.03 mg/mL, and *D*_*t*_ = 30 s (representing a midpoint region of the optimized parameter space highlighted with the cross in Figure 4c). This set of parameters represents one of the many optimal EPD method conditions predicted by the model, and if necessary, further refinement within this region could be relatively easily conducted with only a minimal number of further experiments.

### Sensing Performance of Pt/CNT-Coated Pt-MEs and CFMs

The validity of the DoE-optimized EPD conditions was tested by using a Pt/CNT@Pt-ME (produced under the optimized modification conditions *V* = −4 V, *D*_*t*_ = 30 s, and *C* = 0.03 mg/mL) for electrochemical H_2_O_2_ sensing in phosphate buffer solution (PBS). A calibration plot was produced using CV in the presence of H_2_O_2_, where the current at +600 mV (the oxidation potential of H_2_O_2_) was plotted as a function of the H_2_O_2_ concentration (Figure 5a,b). The gradient of the plot corresponds to the sensitivity of the electrode (Table 2).

**Table 2:** LOD and Sensitivity Values Are Compared for the Optimum EPD Conditions Selected during the DoE and for Two Sets of Conditions at Nonoptimal Regions of the DoE Parameter Space

	EPD modification conditions			H_2_O_2_ sensing performance	
	*V*	*C*	*D*_*t*_	LOD, μM	sensitivity, nA mM^–1^
insufficient Pt-ME coating (DoE red band)	-4	0.01	10	97.8	8
DoE-optimized conditions (DoE orange band)	-4	0.03	30	2.4	9
thicker Pt-ME coating (DoE blue band)	-4	0.05	10	5.1	7

**Figure 5:**
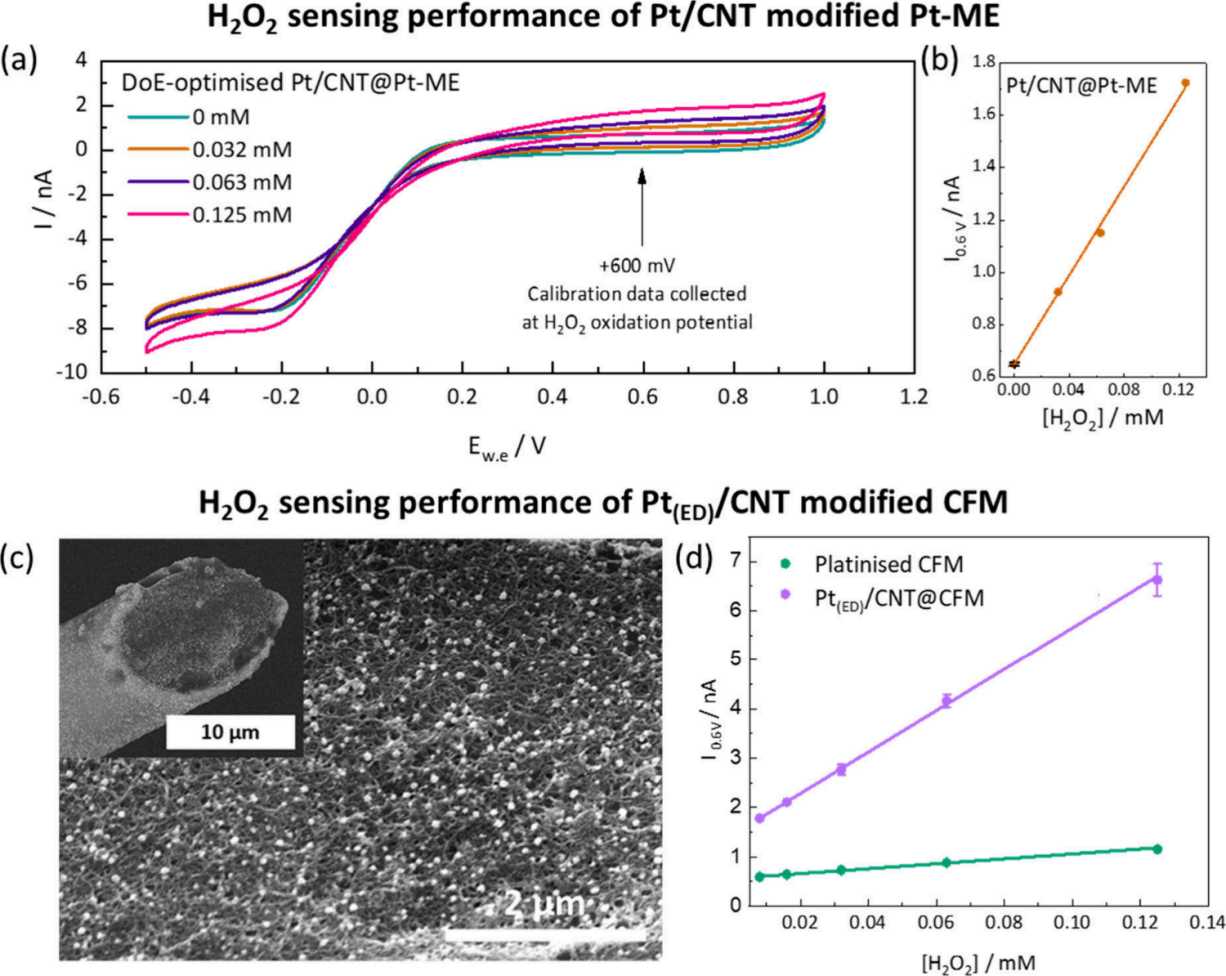
Sensing performance of DoE-optimized Pt/CNT@Pt-ME vs Pt/CNT@CFM: (a) CVs for H_2_O_2_ in PBS (pH 7.4) for modified Pt-ME (vs Ag/AgCl, 100 mV/s scan rate); (b) H_2_O_2_ calibration plot for modified Pt-ME, based on CV response at +600 mV, 100 mV/s scan rate, as shown in (a); (c) SEM image of Pt/CNT coating on the CFM surface, where inset shows uniform coating across the entire CFM surface; (d) H_2_O_2_ sensing performance of Pt_(ED)_/CNT@CFM compared to a platinized CFM, using CVs at 100 mV/s scan rate.

The resulting Pt/CNT@Pt-ME showed an LOD of 2.4 μM for the detection of H_2_O_2_ (Table 2), well within the range required for *in vitro* biological H_2_O_2_ measurements.^8,30,53,54^ This contrasts with poorer H_2_O_2_ sensing performance for Pt-MEs modified using conditions identified as less desirable from the DoE studies (calibration curves in Supporting Information Figure S12). For example, Pt-MEs modified at −4 V, 0.01 mg/mL, and 10 s (red band) showed a substantial deterioration of the H_2_O_2_ LOD (which increased by almost 2 orders of magnitude to 97.8 μM). Pt-MEs modified under comparatively high Pt/CNT concentration conditions (0.05 mg/mL, −4 V, 10 s, blue band) also showed poorer H_2_O_2_ sensing performance, with a poorer LOD (increase by a factor of 2).

Interestingly, the sensitivity of H_2_O_2_ sensing is considerably less impacted by the variation in EPD conditions within the parameter space, likely reflecting the overall relatively thin deposits created in the parameter space studied, as well as the H_2_O_2_ sensing background activity of the underlying Pt-ME surface. Sensitivity improvements are in fact relatively moderate (∼50%) compared to the unmodified Pt-ME. However, crucially, LoD improved significantly compared to the unmodified Pt-ME, decreasing from 25 to 2.4 μM, an order-of-magnitude enhancement. It should be noted that the sensing results in this study are based on a single electrode functionalization experiment. The clear improvements and trends of the findings do however highlight the efficacy of DoE-based optimization of ultramicroelectrode modification for substantially improving LOD for H_2_O_2_ sensing and for guiding future optimization with replications.

A final set of experiments investigated whether the EPD modification conditions, identified via the DoE methodology described above, can be readily transposed to other ultramicroelectrode systems. First, an alternative EPD modification protocol for Pt-MEs was explored. All studies discussed so far were based on EPD of pre-formed Pt/CNT composites onto the Pt-ME surface. As an alternative approach, a sequential modification method, was also tested. To this end, Pt-MEs were first modified with CNTs only (via EPD at the DoE optimized conditions) to minimize aggregation issues that can occur for pre-formed Pt/CNT composites. The resulting CNT coating was then decorated with Pt nanoparticles in a second step via an electrochemical deposition approach, forming Pt_(ED)_/CNT. Using the Pt_(ED)_/CNT method readily yielded modified Pt-MEs with good H_2_O_2_ sensing performance in a single attempt (LOD = 5.1 μM, sensitivity = 7 nA mM^–1^, Table 3). It is hypothesized that the slightly higher LOD compared with the original methodology arises from greater inhomogeneity of the electrochemically formed Pt nanoparticles (Figure S13).

**Table 3:** Comparisons of LOD and Sensitivity for the Two Ultramicroelectrode Types Used in the Study and the Two Different Pt/CNT Composite Formation Methods

ultramicroelectrode system	abbreviation	LOD, μM	sensitivity, nA mM^–1^
Pt-ME modified via EPD of preformed Pt/CNT composite	Pt/CNT@Pt-ME	2.4	9
Pt-ME modified via EPD of CNTs, followed by Pt electroplating	Pt_(ED)_/CNT@Pt-ME	5.1	7
CFM modified via EPD of CNTs, followed by Pt electroplating	Pt_(ED)_/CNT@CFM	4.4	42

Finally, the DoE-optimized conditions were employed for the modification of a different type of ultramicroelectrode, namely, for the modification of CFMs with Pt/CNT coatings. CFMs are an important ultramicroelectrode class as they are widely used for *in vivo* sensing applications.^14,30,53^ As they are carbon-based, they are often less responsive toward H_2_O_2_ without further modification when using classical CV at slow scan rates, as we show in this work. The CFMs used here have a similar disk size and geometry to the Pt-MEs, making them a valuable comparison.

Specifically, the sequential EPD and electrochemical deposition protocols outlined above to form a Pt_(ED)_/CNT coating were employed. This protocol was chosen as the sequential approach consistently yielded coatings with superior adhesion and stability in aqueous environments, compared to functionalization with presynthesized Pt/CNT, and as the Pt-ME experiments (Table 3) had shown that both functionalization strategies yield comparable LOD and sensitivity. The resulting Pt_(ED)_/CNT@CFM showed again excellent H_2_O_2_ sensing performance (achieved without the need for any additional modification optimization), exhibiting an LOD of 4.4 μM, coupled with a sensitivity of 42 nA mM^–1^ (Table 3). This excellent performance suggests that the EPD conditions developed for the Pt-ME system are directly applicable to another distinct ultramicroelectrode system. This finding is further illustrated by SEM imaging of the Pt_(ED)_/CNT@CFM which shows a uniform monolayer coating (Figure 5c). The beneficial incorporation of the CNTs into the CFM coating was demonstrated through comparison to a platinized CFM, a widely used CFM modification approach in literature,^8,30^ which had an order of magnitude lower sensitivity of 5 nA mM^–1^ and an LOD of 2.9 μM (Figure 5d).

## Conclusions

This work applied DoE screening methodologies to improve the coating of a nanoparticle-based composite onto an ultramicroelectrode substrate via EPD. A simple 2^*k*^ factorial screening DoE method enabled the systematic investigation of a large parameter space in a small number of experiments. A simple yet informative response metric (*I*) and using a three-factor DoE, the impact of key EPD modification parameters on the *I* response (as proxy for ultramicroelectrode coating quality) was modeled using an interaction mathematical model. Following this, a further two-factor DoE enabled additional refinement of the EPD parameter combinations used to give thin, homogeneous, noncharging electroactive coatings.

The resulting modified Pt-ME was used in a model H_2_O_2_ sensing study, where it was shown that the LOD could be significantly improved upon the use of the DoE optimized conditions. The optimized conditions were also translated to an alternative, carbon-fiber-based ultramicroelectrode (CFM) and used with a different composite formation method (Pt_(ED)/_CNT), enabling highly successful ultramicroelectrode modification in a single experiment.

Comparison of the resulting LOD values to the literature is challenging as there have been many different modification methods used to coat ultramicroelectrodes; however, the LODs achieved using the DoE in this work are in line with similarly modified ultramicroelectrodes implemented *in vitro* biological sensing applications.^8,30,53,55,56^ Future work will expand the characterization of the functionalized ultramicroelectrodes and elucidate the correlation between the electrochemical performance and coating thickness. It will also enable an in-depth characterization of the limit of detection and the detection reproducibility of different analytical targets. We will also employ alternative DoE target metrics such as the oxidation current of H_2_O_2_ or the ultramicroelectrode sensitivity.

Through the use of DoE principles and the mapping out of the corresponding design space, this work outlines a data-driven, systematic, and easily repeatable approach to ultramicroelectrode modification. The DoE approach provides key benefits over more conventional OVAT methodologies, e.g., by providing insights into interdependencies between multiple modification parameters, such as concentration, deposition time, and voltage, via a small number of experiments. Importantly, the DoE approach yields not a single optimized parameter combination but clearly indicates a wide range of suitable EPD parameters while allowing identification of limiting conditions where EPD ultramicroelectrode modification becomes less effective. As such, the methodology presented here allows for the development of more robust and repeatable ultramicroelectrode modification processes and assessment of the “process tolerances”, crucial for wider adoption of ultramicroelectrode modification strategies and future technology translation. DoE data fitting via more complex models, potentially based on known, EPD-related relationships, such as the Hamaker equation, could be explored to improve accuracy of the modeled response surfaces and explore interactions between EPD parameters further. Other mathematical models and DoE approaches could also open up opportunity for optimization using more advanced algorithms, potentially via methods that allow for more automated readout and optimization, aiding technological development and large-scale manufacturing of modified micro- and nanoelectrodes in the future.

## Supplementary Material



## Data Availability

Raw data are available from the authors on request. DoE MatLab script is available free of charge at https://github.com/tombom3000000/NanoparticleModificationOfMicroelectrodesDOE.

## Notes

Additional Pt/CNT composite and Pt-ME characterization; additional DoE data, DoE designs and model validation data (PDF)
