# Supplementary material for: Nanomaterial Modification of Ultramicroelectrodes Using Design-of-Experiments Principles
Source: ACS Electrochem. 2025 Nov 27;2(1):101–12. doi: 10.1021/acselectrochem.5c00227 (PMC12766678; doi:10.1021/acselectrochem.5c00227)
Supplement: Supplementary file 1 [file ec5c00227_si_001.pdf]

Supporting Information

Nanomaterial Modification of Ultramicroelectrodes using Design-of-Experiments Principles

Rachel A. Bocking [1,2], Thomas M. Dixon [1,3], Brenna Parke [4], Parastoo Hashemi [4], Richard A. Bourne [1,3], Paolo Actis [2,5] and Robert Menzel* [1,2]

Corresponding author email: r.menzel@leeds.ac.uk

1. School of Chemistry, University of Leeds, Leeds LS2 9JT, United Kingdom; 2. Bragg Centre for Materials Research, University of Leeds, Leeds LS2 9JT, United Kingdom; 3. Institute of Process Research and Development; University of Leeds, Leeds LS2 9JT, United Kingdom; 4. Department of Bioengineering, Imperial College London, London SW7 2AZ, United Kingdom; 5. School of Electrical and Electronic Engineering, University of Leeds, Leeds LS2 9JT, United Kingdom

Figure S1. Characterization of Pt/CNT composite (PXRD, TGA, XPS).

Figure S2. TEM of Pt/CNT composite.

Figure S3. SEM of Pt-ME with dip-coated surface.

Figure S4. Pore segmentation analysis of SEM images showing coated electrode surfaces.

Table S1. Porosity analysis of pore segmentation data.

Table S2. Three-factor DoE data.

Figure S5. Alternative DoE model matrix plot.

Figure S6. Alternative Two-factor DoE model design space.

Figure S7. Alternative Two-factor DoE with validation points.

Figure S8. Three-factor DoE design space model.

Figure S9. SEM and CV of unmodified Pt-ME.

Table S3. Two-factor DoE data.

Figure S10. Two-factor DoE with validation points.

Figure S11. Predicted vs. actual plots for I_{Ruhex} response.

Figure S12. Calibration plots for H_2O_2 sensing using CV.

Figure S13. SEM and TEM of $Pt_{(ED)}$/CNT with particle size distribution.

Table S4. Confidence intervals for three-factor DoE model.

Table S5. Confidence intervals for two-factor DoE model.

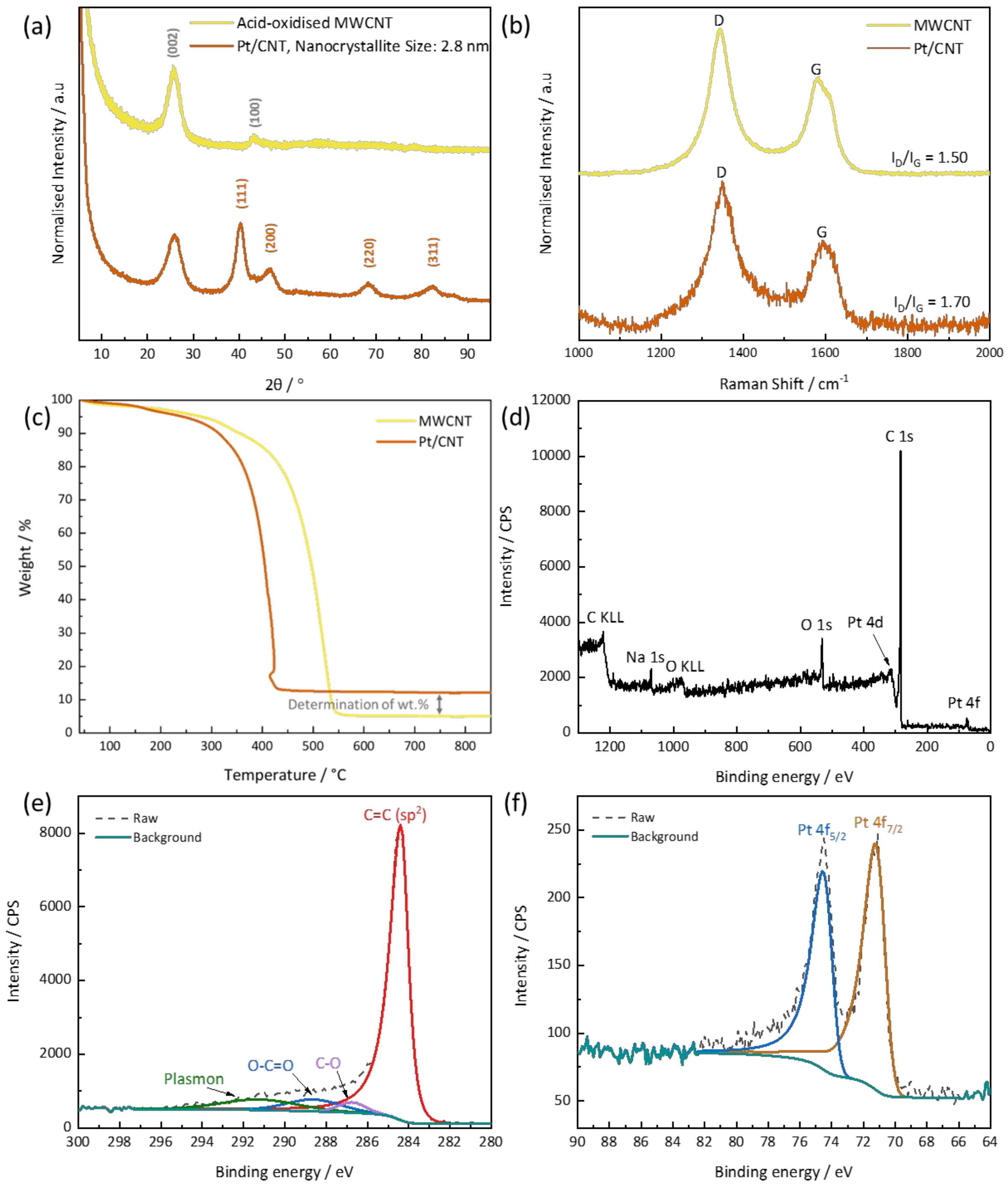

Figure S1. (a) PXRD analysis of the Pt/CNT composite compared to the acid-oxidized MWCNT precursor; (b) Raman spectra for acid-oxidized MWCNT and Pt/CNT; (c) TGA of acid-oxidized MWCNT and Pt/CNT powders in air, highlighting residual metal content to establish wt.% platinum loading; (d) Survey XPS spectrum of Pt/CNT; (e) High-resolution XPS spectrum for acid-oxidized MWCNT; (f) High-resolution XPS spectrum for Pt/CNT.

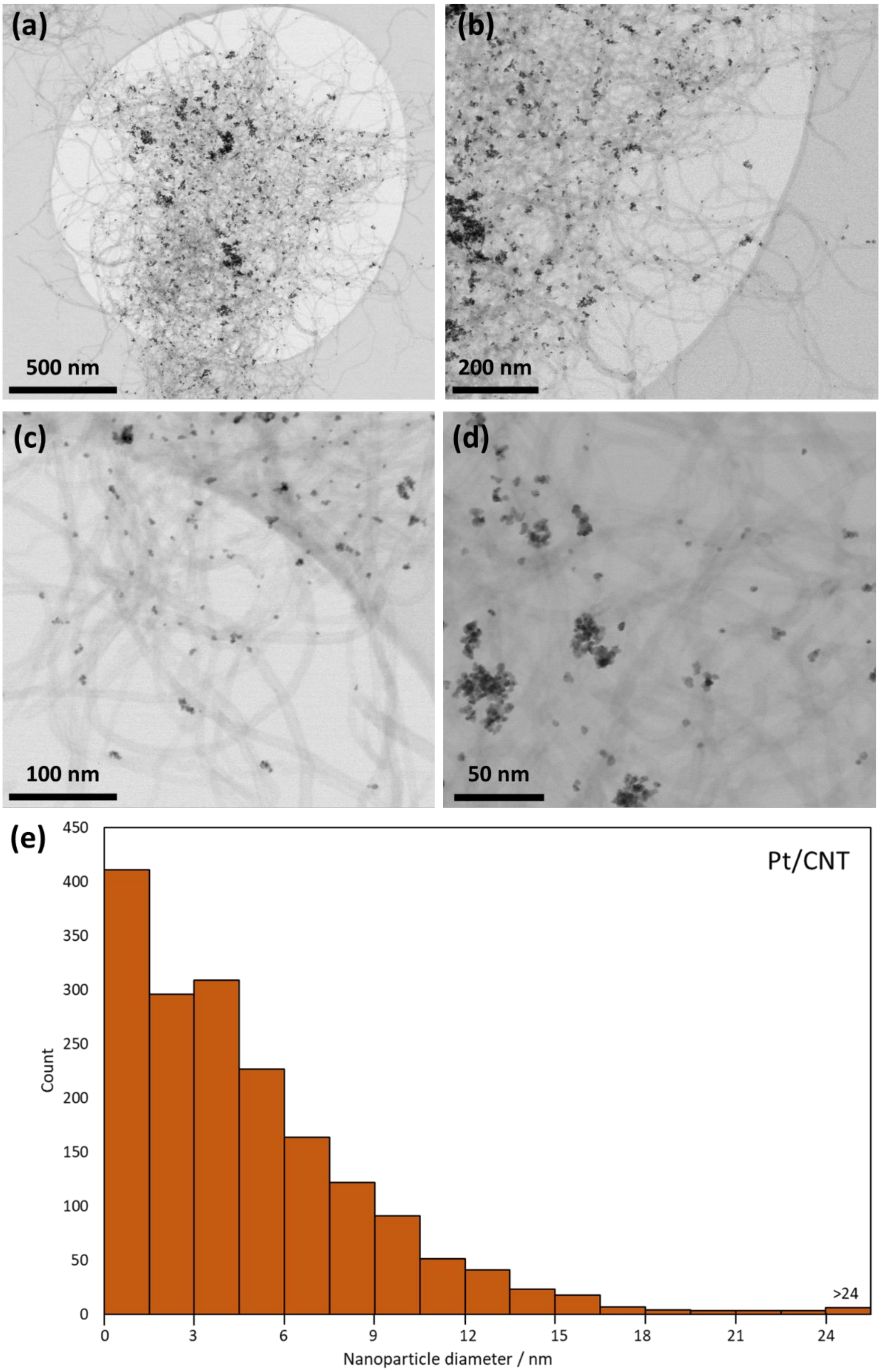

Figure S2. (a-d) TEM images of the Pt/CNT composite powder, highlighting the distribution, size and morphology of the platinum nanoparticles; (e) particle size distribution of platinum nanoparticles as determined through particle sizing of 1779 particles across 7 TEM images.

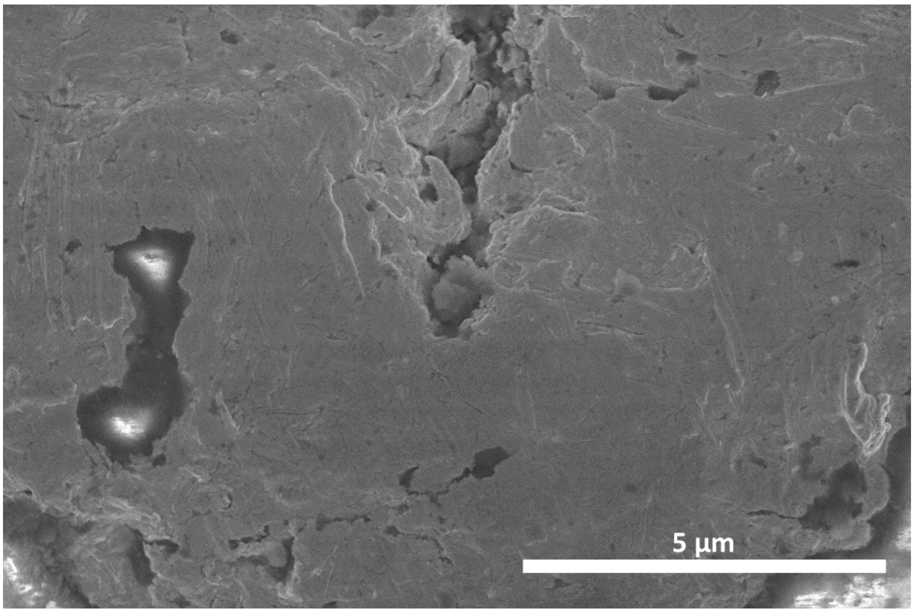

Figure S3. Secondary electron mode SEM image (10,000x) of a Pt-ME surface following EPD of Pt/CNT in DMF at D_t = 5 s, V = -4 V, C = 0.01 mg/mL, highlighting that that very short deposition times are unsuitable for producing a coating.

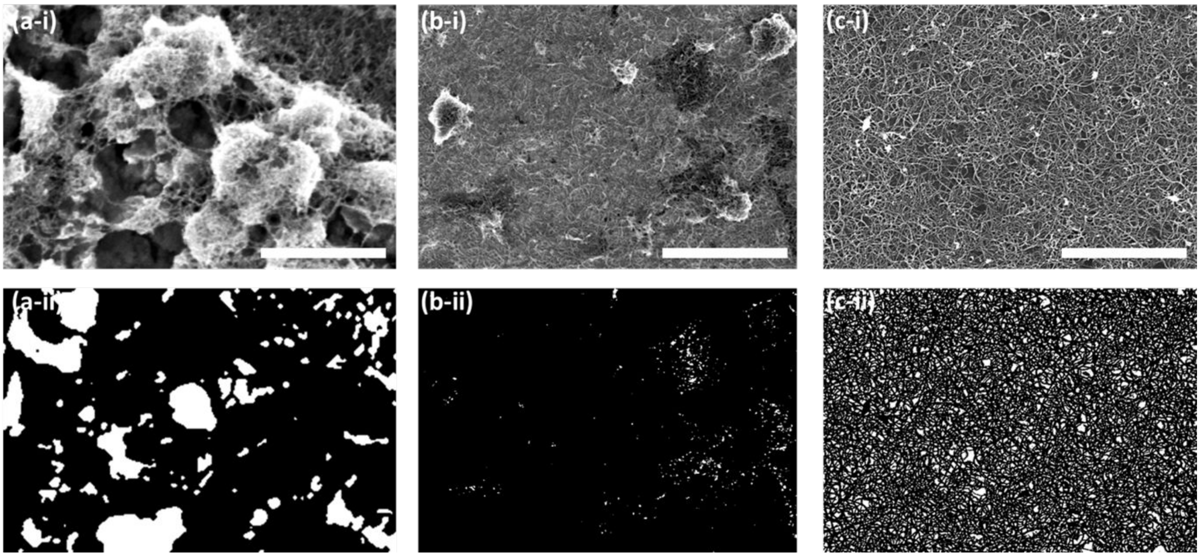

Figure S4. Pore segmentation analysis of SEM images from Figure 2 (main text). SEM images (top row) and mapped 2D pore segments from threshold segmentation analysis (bottom row) of: (a-i & a-ii) thick, multi-layer coating of Pt/CNT; (b-i & b-ii) medium thickness Pt/CNT coating with some agglomerations of CNTs; (c-i & c-ii) thin, homogeneous Pt/CNT coating. Scale bars = 2 µm (same scale for corresponding top and bottom images).

Table S1. Porosity and capacitance data for the coatings shown in Figure 2 in the main text. Porosity analysis data were obtained through threshold segmentation analysis (Figure S4) of Pt/CNT SEM images from Figure 2. The capacitance was estimated from the separation between the forward and reverse scans prior to the Faradaic response (at -50 mV vs Ag/AgCl). Assuming that the entirety of the current in this region is due to capacitive contributions, the capacitance values for the CVs in Figure 2 have been calculated using the method as described in Anderson et al.[1] and is given as mean value of three repeats (n=3). This capacitance includes both double-layer charging and any stray capacitances in the measurement system

Coating	Average Pore Diameter / nm	Pore Count	Pore density / pores per μm^2	Capacitance / nF
Fig 2(a)	200 ± 210	112	6.4	250
Fig 2(b)	40 ± 30	413	13.2	60
Fig 2(c)	30 ± 20	9316	368	20

*Pore diameter refers to the max Feret diameter per pore calculated during image analysis in Image-J software.

Additional Coating Characterization via SEM image analysis

The SEM images of the coatings in Figure 2 of the main text were analysed through pore segmentation analysis. This automated image analysis approach allows to sample 100s to 1000s of macro-pores in one analysis, providing robust data for porosity analysis from electron microscopy imaging. Based on this analysis, the coating in Fig 2(a) shows a relatively small number of pores which are comparatively large and non-uniform (as indicated by the large standard deviation). In contrast, across a similar imaging area, the coating in Fig 2(c) exhibits, a dramatically increased number of pores with smaller average pore size and higher uniformity (i.e. as indicated by the reduced standard deviation). As expected, the coating in Fig 2(b) exhibits an intermediate level of pore density and uniformity. More broadly, the SEM-based porosity analysis indicates that coating porosity becomes more regular, uniform and frequent from Fig 2(a) to Fig 2(c) – contributing to the improved, more repeatable CV characteristics of the thin, target coatings, observed in Fig 2(c).

Methodology for SEM image analysis

Microscopy images were analysed using Fiji-Image J software (version 1.54p). Pores were segmented across the whole image area using thresholding segmentation analysis. The threshold was set (using MinError) to 30%, ensuring that pores were well defined and separated (kept consistent across images), allowing to resolve pores of around 40 nm size or larger. The segmented image was then smoothed to remove small pixel debris.. Very large pores were analysed using a built-in particle analysis tool (particle analysis conditions: Size min 2-infinity pixel units, circularity 0-1, include holes). Image analysis gave the values reported in the table, including the average pore diameter (the mean of the maximum Feret diameters of the individual pores), pore count (the number of pores in the image area) and the pore density (the ratio of pore count over image area).

Table S2. Full experimental data for the three-factor DoE, including the I_{Ruhex} response for all 11 experimental points.

Experiment number	Applied EPD Voltage / V	EPD duration /s	Pt/CNT Concentration / mg ml^{-1}	I_{ruhex} Response/ nA
1	-4	10	0.01	-35.0
2	-4	10	0.05	-115.8
3	-4	60	0.01	-32.0
4	-4	60	0.05	-87.6
5	-4	60	0.05	-58.8
6	-4	60	0.05	-74.0
7	-1	10	0.01	-58.9
8	-1	10	0.05	-44.5
9	-1	60	0.01	-57.3
10	-1	60	0.05	-86.2
11	-2.5	35	0.03	-60.7

Alternative model fitting

The data was also fitted to an interactions model where the I_{Ruhex} response was multiplied by -1 and then log-transformed, for both the 2^3 and the 2^2 design spaces. For the 2^3 design space, this gave the relationship shown in Equation S1, which had an R^2 of 0.84 and an RMSE of 0.24:

$$\ln(I_{Ruhex} * -1) = 3.71 + 0.23\boldsymbol{V} - 0.38\boldsymbol{D_t} + 0.90\boldsymbol{C} + 0.65\boldsymbol{VD_t} - 0.89\boldsymbol{VC} + 0.10\boldsymbol{D_tC} \quad (S1)$$

This data shows an improved R^2 and RMSE compared to the standard interactions model outlined in Equation 4 in the paper. The general trends shown by both models have many similarities and highlight similar regions of the matrix plot (Figure S5) where optimal results (orange regions) lie. However, this modified interactions model was not used in the work outlined in the paper as the goal was to implement a simple and easy-to-understand DoE model that simplifies the parameter effects on I_{Ruhex}.

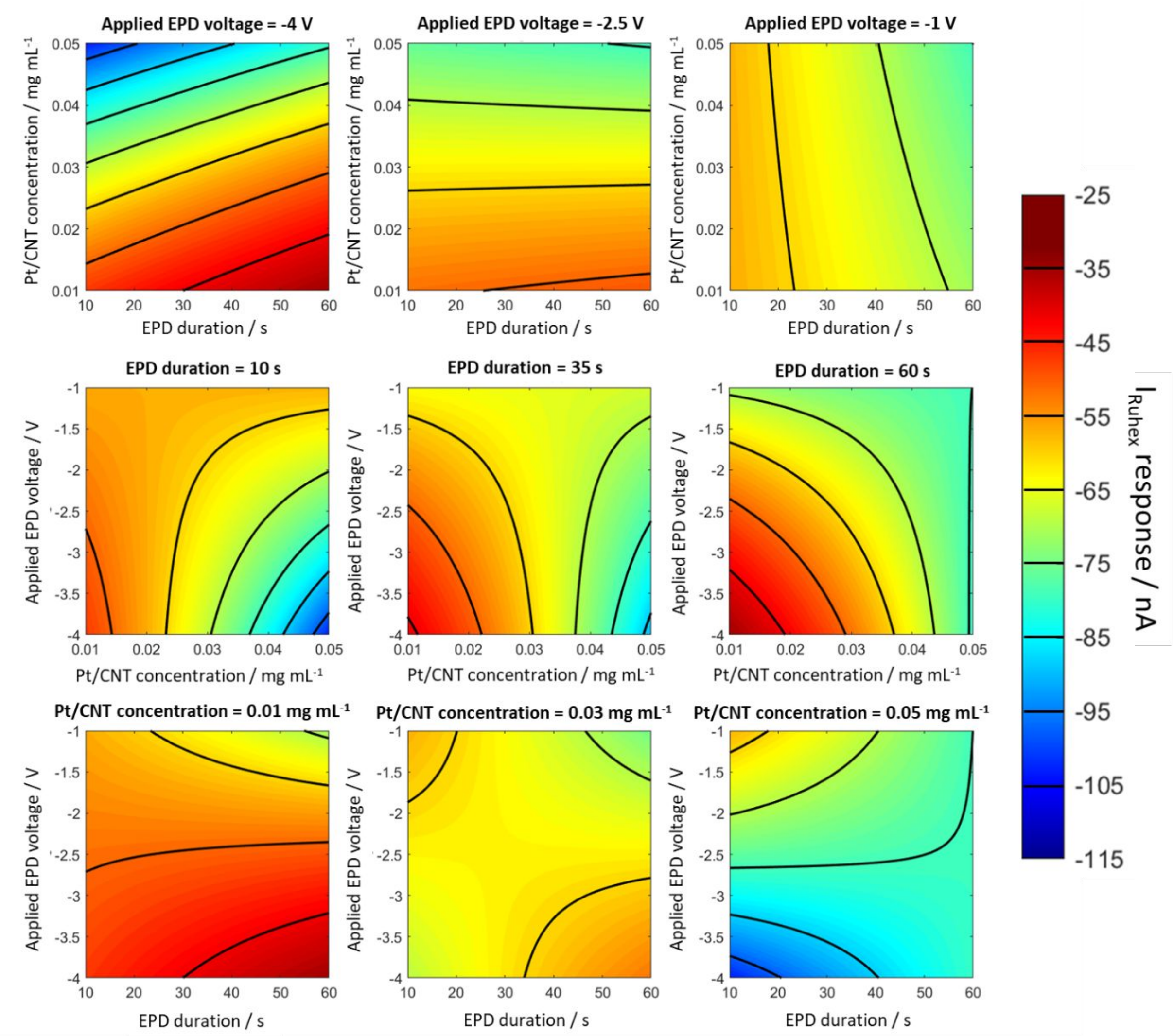

Figure S5. A matrix of contour plots at various combinations of *V*, D_t and *C* within the parameter space modelled by Equation S1, highlighting different regions of the parameter space for I_{Ruhex} responses based upon the interactions model with log transformation.

Similarly, the same interactions model with log transformation was fitted to the 2^2 DoE design space. This yielded an R^2 of 0.85 and an RMSE of 0.21, with the model shown in Equation S2:

$$\ln(I_{Ruhex} * -1) = 3.45 - 0.01\boldsymbol{D_t} + 0.96\boldsymbol{C} - 0.13\boldsymbol{D_t}\boldsymbol{C} (S2)$$

The model again is similar to the 2^2 interactions model outlined in the paper in Equation 2. However, the optimal response region boundaries were shifted to a slightly higher *C* than with the standard interactions model.

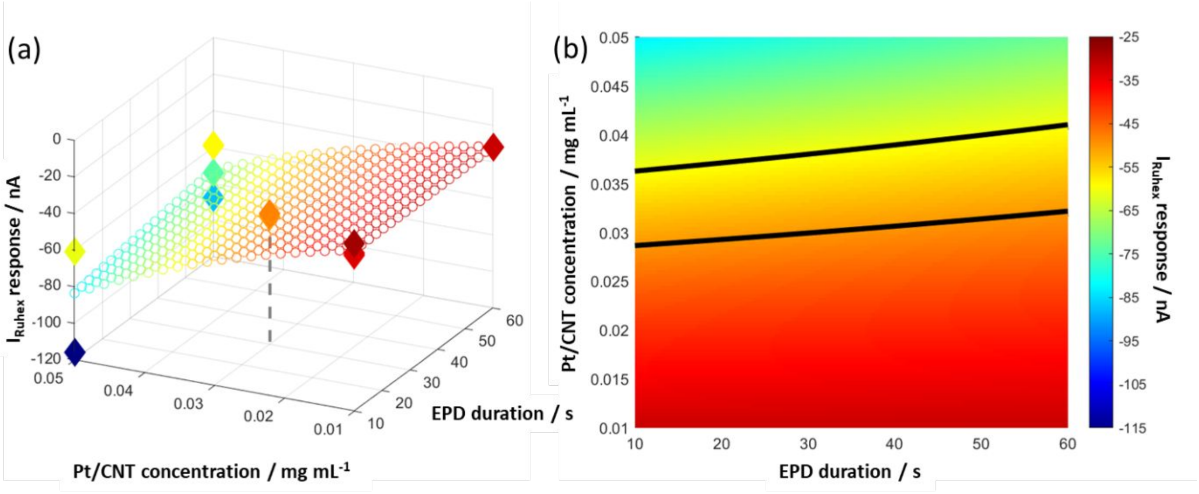

Figure S6. (a) the two-factor design space with the I_{Ruhex} plotted as the z-coordinate to show repeatability of data, along with the response surface defined in Equation S2; (b) the response surface defined in Equation S2 where the region within the black boundary lines highlights suitable parameter combinations that would yield an I_{Ruhex} response in the region -55±5 nA.

The coefficient terms also agree with the model outlined in the paper where the interaction between D_t and C is small, suggesting they behave independently. The C coefficient is large, suggesting the I_{Ruhex} response is mainly dominated by C effects.

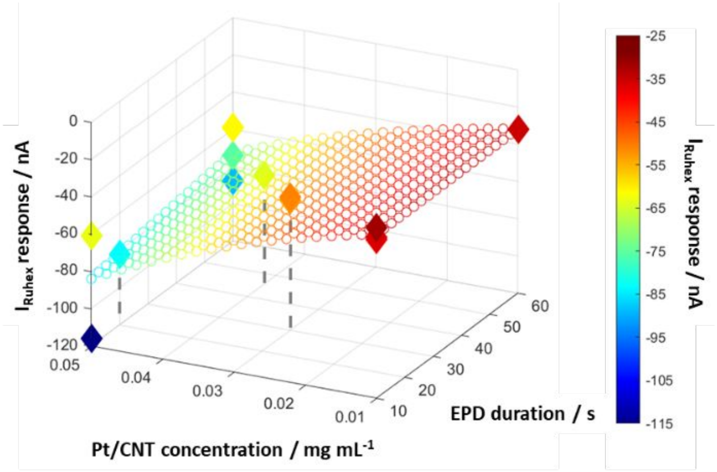

Figure S7. 3D representation of the two-factor DoE with added points highlighting the good fit of the new data points to the interactions model with log transformation which is represented by the hollow coloured circle layer cutting through the design space.

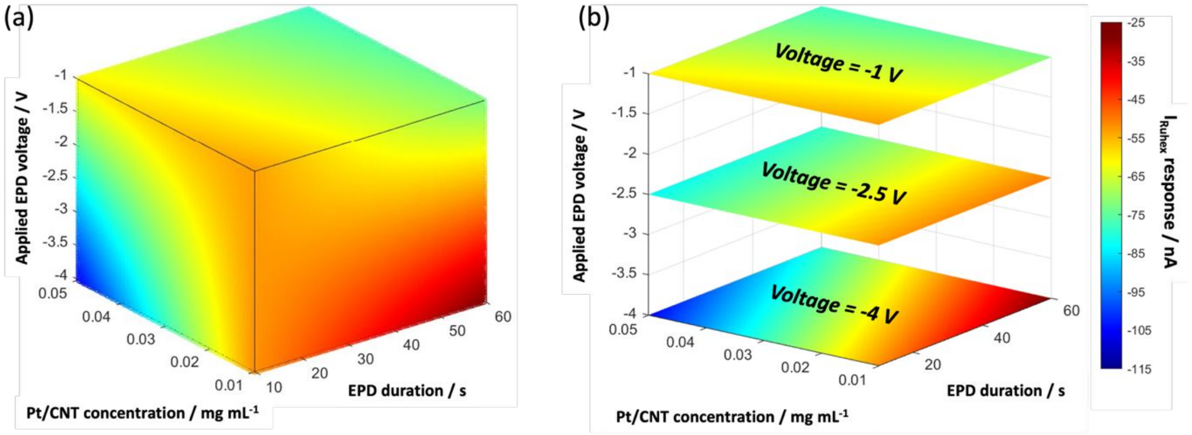

Figure S8. (a) The three-factor DoE design space as shown in Figure 1(c) is here sliced into three planes at -1 V, -2.5 V and 4 V, as shown in (b).

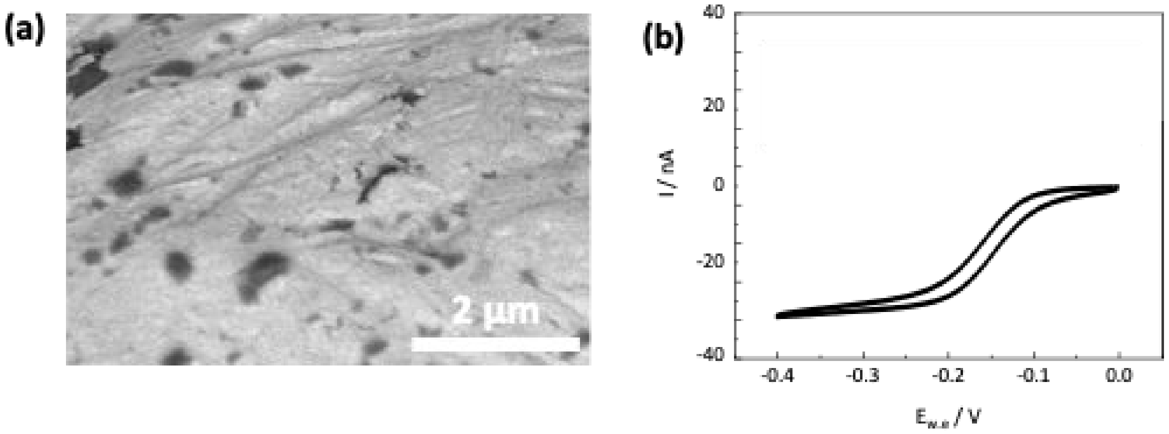

Figure S9. SEM image and respective I_{Ruhex} CV curves for an unmodified 15 µm in diameter Pt-ME. CV was carried out in 10 mM $Ru(NH_3)_6Cl_3$ in 0.1 M KCl and the current at 400 mV was taken as I_{Ruhex}. The CV method parameters consisted of: E_i = 0 V vs. Ag/AgCl Ref., Scan rate = 100 mV/s, E_1 = -0.4 V vs. Ref., E_2 = 0 V vs. Ref., nc = 4, 50 % step duration, N = 10 voltage steps.

Table S3. Full experimental data for the two-factor DoE, including the I_{Ruhex} response for all 11 experimental points.

Experiment number	EPD duration /s	Pt/CNT Concentration / mg ml^{-1}	I_{ruhex} Response/ nA
1	60	0.05	-87.6
2	60	0.05	-58.8
3	60	0.05	-73.9
4	60	0.01	-32.0
5	10	0.01	-35.0
6	10	0.01	-28.5
7	10	0.01	-33.5
8	10	0.05	-115.8
9	10	0.05	-60.9
10	30	0.03	-50.3
11	30	0.03	-48.4

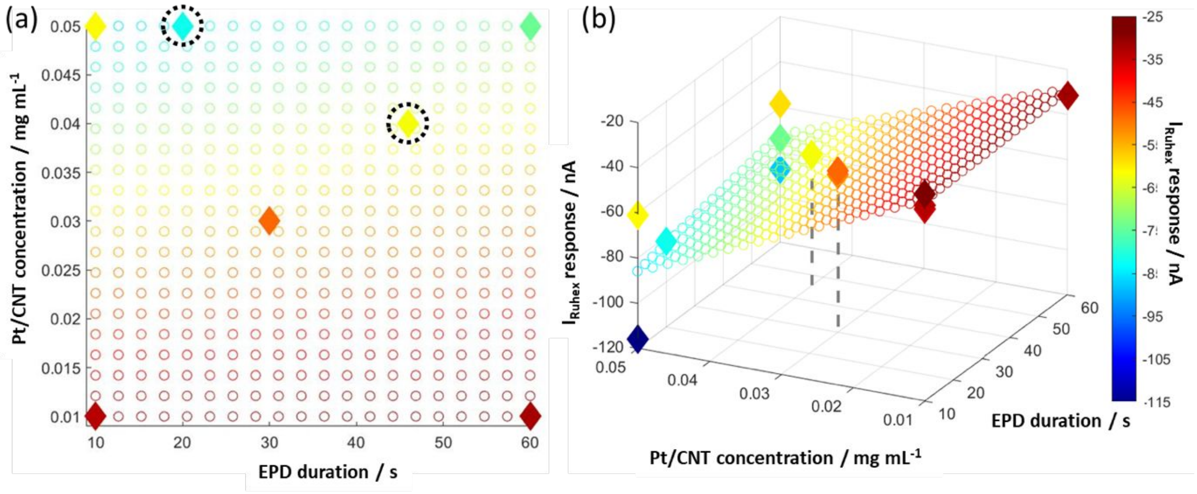

Figure S10. (a) Added points in the two-factor DoE highlighted with black rings; (b) 3D representation of the two-factor DoE with added points highlighting the good fit of the new data points to the interactions model which is represented by the hollow coloured circle layer cutting through the design space.

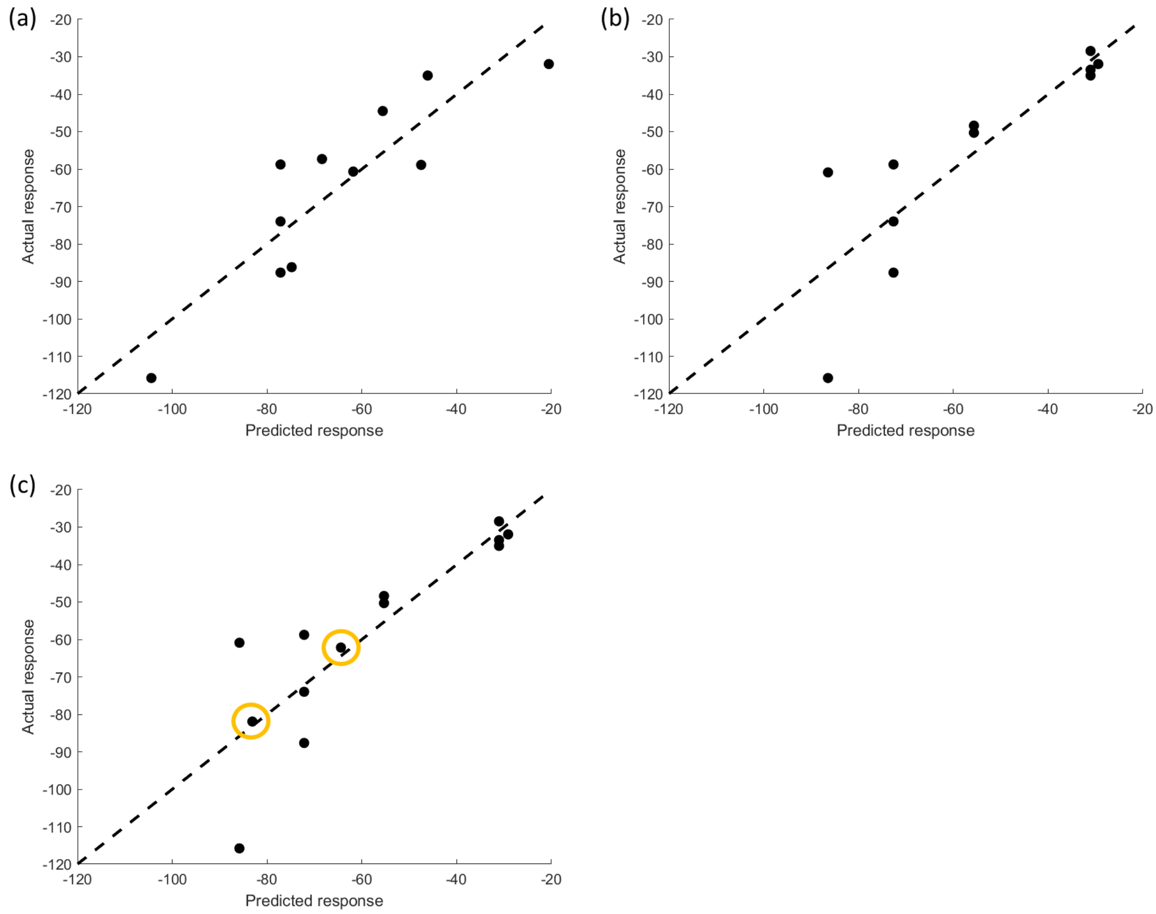

Figure S11. Predicted vs. actual plots for the I_{Ruhex} response, where the predicted response is that predicted by the interactions model; (a) 3D DoE dataset; (b) 2D DoE dataset; (c) 2D DoE dataset with the added validation points highlighted.

The measured I_{Ruhex} response values for the three-factor DoE, two-factor DoE, and two-factor DoE including validation points are plotted versus the response values predicted by the interactions model for the 3D and 2D DoE datasets to visualize the robustness of the model, especially at conditions yielding a low to moderate response value.

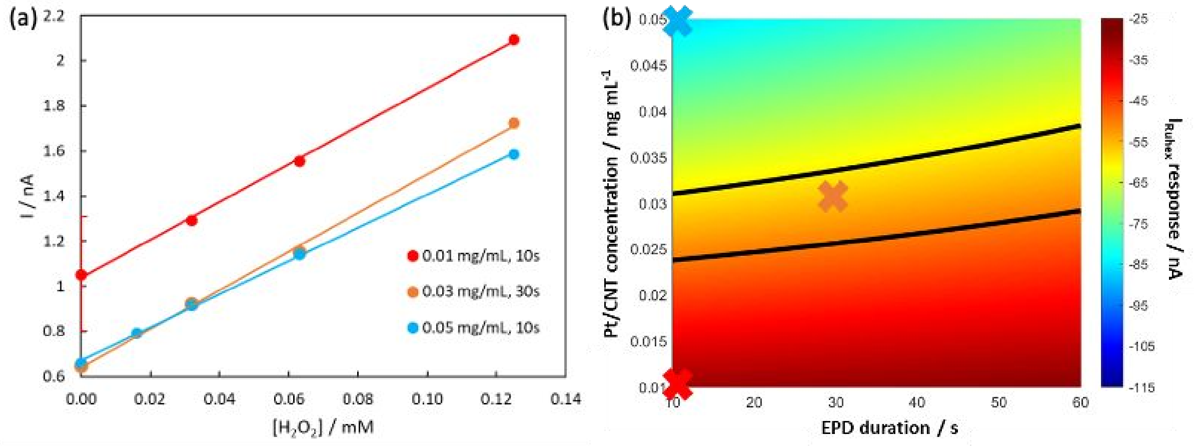

Figure S12. (a) Calibration plots for H_2O_2 sensing for three points in the design space; standard deviations (n=3) for 0 mM H_2O_2 data points: σ(0.01mg/mL, 10s) = 0.273 nA; σ(0.03mg/mL, 30s) = 0.007 nA; σ(0.05mg/mL, 10s) = 0.05 nA; root mean square error of fit: RMSE(0.01mg/mL, 10s) = 0.058; RMSE(0.03mg/mL, 30s) = 0.0086; RMSE(0.05mg/mL, 10s) = 0.0036); (b) the two-factor design space populated with crosses indicating the corresponding regions of the design space to the calibration plots in (a).

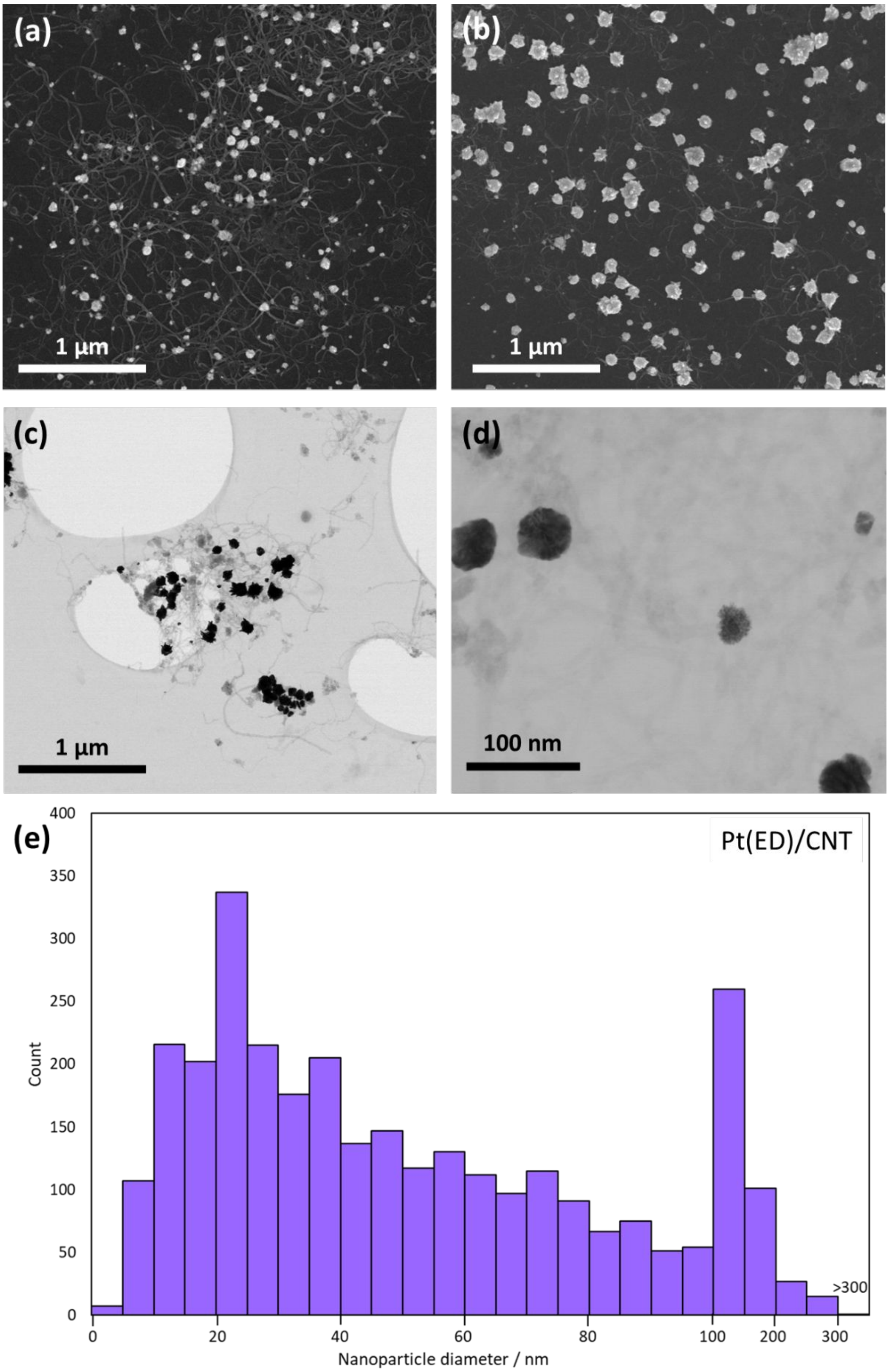

Figure S13. (a-b) SEM images of $Pt_{(ED)}$/CNT on glassy carbon using a secondary electron detector at 25000x magnification. Across different samples there were visible differences between nanoparticle morphologies; (c-d) TEM images highlighting the morphologies of the platinum nanoparticles within the same sample; (e) particle size distribution of platinum nanoparticles in $Pt_{(ED)}$/CNT as formed through electroplating, using 3062 nanoparticles across 7 images.

Table S44. Confidence intervals (95 %) for the three factor DoE model (**Equation** 4).

Variable	Lower bound	Upper bound
Constant	-93	1
V	-63	60
D_t	-36	87
C	-120	3
VD_t	-115	22
VC	-18	119
D_tC	-67	70

Table S55. Confidence intervals (95 %) for the two factor DoE model (**Equation 5**).

Variable	Lower bound	Upper bound
Constant	-54	-8
C	-45	48
D_t	-92	-19
CD_t	-47	71

References

(1) Anderson, K. L.; Edwards, M. A. A Tutorial for Scanning Electrochemical Cell Microscopy (SECCM) Measurements: Step-by-Step Instructions, Visual Resources, and Guidance for First Experiments. *ACS Measurement Science Au* **2025**, *5* (2), 160-177. DOI: 10.1021/acsmeasuresciau.4c00091.
